# P04.54. Natural health service: enhancing wellbeing with group walks in green spaces

**DOI:** 10.1186/1472-6882-12-S1-P324

**Published:** 2012-06-12

**Authors:** M Marselle, S Warber, K Irvine

**Affiliations:** 1 De Montfort University/Institute of Energy and Sustainable Development, Leicester, England; 2 University of Michigan Integrative Medicine, Ann Arbor, USA

## Purpose

Mental health and wellbeing are increasingly recognized as fundamental to quality of life; at the same time, mental disorders are increasing. In the UK, a quarter of General Practitioner (GP) consultations are about mental health. Alternative programs to address mental health are necessary for patient-centered care. Research suggests that interaction with nature contributes to mental wellbeing. This paper investigates whether participating in a green space walking group – Walking for Health (WfH) – makes a difference to mental health and wellbeing in the general population. An English GP developed WfH as an intervention to promote physical activity by providing free, organised and led group walks in local natural environments across England.

## Methods

A quasi-experimental research design investigated the mental health and wellbeing among adults living in England who had participated in WfH during 2011 (n=1,968) and those who had not (n=1,412). An online questionnaire assessed participants’ mental wellbeing, depression, affect, perceived stress, psychological resiliency, and social support at baseline and 13-weeks follow-up. Independent sample t-tests were used to compare mean scores at baseline. Participants with missing responses on a scale were excluded from that particular analysis.

## Results

Adults who attended WfH walks had significantly greater mental wellbeing (p<0.01; effect size d=0.19), greater positive affect (p<0.01; d=0.11), fewer depressive symptoms (p<0.01; d=0.30), less negative affect (p<0.01; d=0.34), and less perceived stress (p<0.01, d=0.31) than adults who did not participate in WfH walks. The two groups did not differ in protective measures of social support (p=0.525) and resiliency (p=0.811), which could contribute to differential mental health and wellbeing.

## Conclusion

This research demonstrates the value of using organized group walks in the natural environment for positive mental health and wellbeing. Green space group walks are possibly a cost-effective way to improve mental health. They may also be an alternative treatment for common mental health disorders.

